# Machine learning approach for recognition and morphological analysis of isolated astrocytes in phase contrast microscopy

**DOI:** 10.1038/s41598-024-59773-2

**Published:** 2024-04-29

**Authors:** Egor V. Yakovlev, Ivan V. Simkin, Anastasiya A. Shirokova, Nataliya A. Kolotieva, Svetlana V. Novikova, Artur D. Nasyrov, Ilya R. Denisenko, Konstantin D. Gursky, Ivan N. Shishkov, Diana E. Narzaeva, Alla B. Salmina, Stanislav O. Yurchenko, Nikita P. Kryuchkov

**Affiliations:** 1 Scientific-Educational Centre “Soft matter and physics of fluids”, Bauman Moscow State Technical University, 2nd Baumanskaya Street 5, Moscow, 105005 Russia; 2 Research Center of Neurology, 80 Volokolamskoye Shosse, Moscow, 125367 Russia

**Keywords:** Phase-contrast microscopy, Astrocyte

## Abstract

Astrocytes are glycolytically active cells in the central nervous system playing a crucial role in various brain processes from homeostasis to neurotransmission. Astrocytes possess a complex branched morphology, frequently examined by fluorescent microscopy. However, staining and fixation may impact the properties of astrocytes, thereby affecting the accuracy of the experimental data of astrocytes dynamics and morphology. On the other hand, phase contrast microscopy can be used to study astrocytes morphology without affecting them, but the post-processing of the resulting low-contrast images is challenging. The main result of this work is a novel approach for recognition and morphological analysis of unstained astrocytes based on machine-learning recognition of microscopic images. We conducted a series of experiments involving the cultivation of isolated astrocytes from the rat brain cortex followed by microscopy. Using the proposed approach, we tracked the temporal evolution of the average total length of branches, branching, and area per astrocyte in our experiments. We believe that the proposed approach and the obtained experimental data will be of interest and benefit to the scientific communities in cell biology, biophysics, and machine learning.

## Introduction

Astrocytes that are star-like glial cells widely distributed in the central nervous system (CNS), play a crucial role in various CNS functions, including brain development and plasticity, synaptic transmission, blood flow regulation, metabolic control within the neurovascular unit^1^. Particularly, glycolytically active astrocytes ensure neuron-astroglial metabolic coupling meeting the metabolic needs of stimulated neuronal cells, control local microcirculation and permeability of the blood–brain barrier^2^. The role of astrocytes in neuroinflammation and regulation of neurogenesis in also well-known. Thus, it is not surprising that altered astroglial structure and functional activity is always seen in brain injury, stroke, neurodevelopmental disorders and neurodegeneration^1,3–7^. The morphology of astrocytes reflects their metabolic and developmental status^8,9^. The activation of astrocytes is usually associated with the rise in the cytosolic free calcium which triggers the activity or numerous signaling cascades. Some of them result in fast and transient or postponed and long-lasting changes in astroglial morphology required for astroglial adaptation to the microenvironment, migration, and interaction with other cells. Therefore, astroglial morphology is different in various brain regions (e.g. protoplasmic vs fibrous astrocytes), in loci of inflammation (resting vs polarized astrocytes), within the so-called astroglial synticium (when connexon-coupled astrocytes may simultaneously respond to extracelllular stimuli), and in a close vicinity to synaptically coupled neurons or microvessels (more branched astrocytes migth produce more contacts with activated neuronal cells or endothelial cells). Thus, detecting, counting, and tracking astrocyte growth dynamics and their morphology are critical for better understanding astroglial contribution to the regulation of brain functions.

The morphology features of astrocytes can be studied experimentally, for example, using microfluidic chips^10,11^ various in situ^12^ and in vivo^13^ methods, as well as using computer modeling (in silico)^14–22^. Despite the fact that time-reducing experimental methods based on Generative adversarial network were developed^23^, cellular dynamics simulation methods significantly reduce time and costs and could lead to improved experiment quality, enhancing our understanding of processes occurring at the cellular and subcellular levels^24^. Moreover, it enables the study of phenomena that are inaccessible experimentally. Similar situation occurred, for example, in the field of condensed matter physics when computational modeling methods such as classical or quantum molecular dynamics significantly improved our understanding of processes occurring at the atomic level. Computational modeling methods have already been successfully applied for studying blood-brain barrier permeability^21^, creating detailed mesh models for visual analysis^22^, reconstructing and exploring various types of astroglia^15^, astrocytic signal transduction through cytosolic $Ca^{2+}$ dynamics^16^, diffusion-weighted signaling^17^, the impact of different substances (in this case, glutamate) on astrocytic signal transduction^20^, interaction between neuronal and astrocytic networks^18^. Besides, using computer cell modeling^25,26^, it is possible to study tumor growth^27^, study the formation of blood vessels during angiogenesis and vasculogenesis^28–35^. However, any computer model must be validated based on experimental data, and in the case of living systems, this data should arise from various multiomics approaches. The morphology data is one of many types used to verify computer models^17,18^. Considering the significant variability within living systems (such as cellular noise), this data should be averaged across a substantial representative sample. This imposes significant requirements for the scalability of experiments that verify computer models.

For the experimental study of astroglial morphology various types of microscopy are utilized, including phase contrast^36^, electronic^37^, fluorescent^38^, etc. These methods were used to study the interaction of astrocytes within the astroglial network^37,39^, the role of astrocytes in cerebral metabolic interactions^40^, mitochondrial metabolism^41^, differences in ensembles of astrocytes in the cortex^42^ and other brain regions^43^. Microscopy is always used to observe brain cells exposed to the action of neurotransmitters, gliotransmitters, cytokines^44^ or cytotoxic substances^45^ either in vitro or in vivo, as well as in affected brain regions in vivo, e.g. in brain injury or neurodegeneration^46–49^. The role of astrocytes in the formation and maintenance of synapses was also studied using microscopy^50–53^. The most common method of visualizing cells is fluorescence microscopy, that usually requires fixation and staining of cells. This method allows for more accurate visualization of cells and organelles. However, these techniques mainly deal with non-viable cells due to procedure of fixation (excluding the application of protocols with fluorescent probes added to alive cells in culture or in the tissue). As a result, the accuracy of estimation of astroglial structure might be greatly compromised. In the conditions in vitro, the morphology of astrocytes might be additionally affected by their adhesion to the the surface with high stiffness (glass or plastics) and establishment of non-physiological monolayers in 2D cultures, or by their inappropriate positioning within 3D polymers. Therefore, if it is necessary to study the parameters and properties of cells in a long-term experiment and at different points in their life cycle, this method will not be suitable. Another solution is the use of phase contrast microscopy, which does not require cell fixation and staining, but it has a drawback of low-contrast images (compared to fluorescence microscopy), thereby making post-processing challenging. Note that despite the lower resolving power of phase contrast microscopy compared to staining methods, the corresponding data still hold significant value for various research purposes.

At present, various approaches for cell image analysis exist, with one of the most commonly used being machine learning. Particularly, the open-source tool ilastik^54^ is actively used for interactive image classification, segmentation, and analysis. However, although modern machine-learning approaches could handle heterogeneity by combining multiple ML models trained on each single cell sub-track^55^ and using peer prediction paradigm^56^, traditional machine learning methods are not suitable for astrocytes detection due to their complex morphology^57^. It was also demonstrated how to derive deep learning-based features to characterize cell morphodynamics by combining time-lapse microscopy with resilient deep learning software designs^58^. Additionally, methods were outlined showing that these descriptors remain largely unaffected by common sources of artifacts in bioimaging analysis^59^.

Using deep learning methods, especially considering advances of deep convolutional neural networks (DCNN) in pattern recognition and image classification problems^60,61^, would be more appropriate. In last few years, a lot of different DCNN models were created^62–67^, which has been used in different fields, for instance biomedical images analysis^66,68–71^, physisc and chemistry tasks, etc.^72–75^. Thus, DCNN can successfully process complex morphology because of multiple layers of feature extraction that makes it suitable for detection and segmentation^76,77^.

In the present paper, we propose a new deep learning-based approach for recognition and morphological analysis of isolated astrocytes. For this purpose, we conducted a series of experiments involving the culture of primary cultures of rat brain cortex astrocytes, followed by the phase contrast microscopy analysis. We labeled the astrocytes in the acquired images and used this data to train the DCNN-based model. Using the proposed approach, we tracked the time evolution of the average total length of branches, branching, and area covered by an astrocyte. The novelty of the proposed approach lies in its ability to recognize morphological data from unstained cell microscopic images. We believe that the proposed approach and the obtained experimental data will be of interest and benefit to the scientific communities in cell biology, biophysics, and machine learning.

## Results

Astrocyte detection methods with an accuracy (precision P = 0.86) comparable to that of human experts already exist^57,78^. However, these methods were created for the detection of immunohistochemically stained astrocytes in high-contrast images, and are not suitable for our experiments. For this reason, the Mask R-CNN neural network^79^, designed for segmentation^80^, was used to solve the problem of segmentation of living astrocytes. We used a Mask R-CNN model pre-trained on the COCO dataset with Inception Resnet v2^81^ on 1024x1024 pixel images. We chose Mask-RCNN over the popular Segment Anything Model^82^ for the following reasons. Firstly, the pre-trained model Mask R-CNN show high performance when trained in segmentation living cells^83^. Secondly, the authors^84^ showed that they were able to outperform the segmentation accuracy of the latest SAM model using improvements to the existing MASK-RCNN model. Thirdly, it was concluded^85^ that SAM’s zero-shot segmentation performance is considerably inferior to that of traditional deep learning-based methods. Thus, the selection of the most accurate segmentation model with the fitting of hyperparameters deserves a separate study.

The Mask-RCNN model can process images up to a resolution of 1024 by 1024 pixels. However, to reduce computational efforts, we used resized images for model training. The final size of the input images is shown in Table 1.

**Figure 1:**
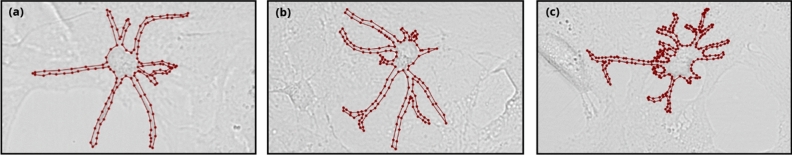
Example of a labeled image of an astrocyte from the rat brain cortex obtained with phase contrast microscopy in our experiments.

The neural network was trained on images labeled using LabelMe^86^; examples are shown in Fig. 1. For training, microscopic images were pre-processed. They were divided into fragments of 1024x1024 pixels (160x160 $\mu$m), with each fragment containing up to 6 astrocytes. The image size of 1024 $\times$ 1024 pixels for raw images determined by equipment (video camera). Fragments in which astrocytes were not detected were discarded. As a result, we obtained 221 images of 1024 $\times$ 1024 pixels, containing in total 332 labeled astrocytes recorded on different days of time evolution. All labeled images were divided into three datasets: 75% were included in the training dataset, 15% in the validation dataset, and 10% in the test dataset. To improve the detection of astrocytes^87^, the training and validation dataset were artificially expanded using the following methods: (i) X-axis flip, random adjustment, (ii), contrast, (iii) brightness, and (iv) random cropping. The dataset size increased to 1105 images by augmentation.

We utilized the acquired datasets to train three models, and the corresponding information is summarized in Table 1. For training the following parameters were adjusted: total steps, warm steps, image resizing. The main difference among the models lay in the resolution of the resized images used during training: (i) Model 20 correspond to 256 $\times$ 256 px, (ii) Model 30 correspond to 512 $\times$ 512 px, and (iii) Model 50 correspond to 700 $\times$ 700 px. We found that Model 50 achieved the highest segmentation accuracy (dice coefficient) on the test set (see Table 1). This is attributed to the high resolution of the input images used for training. However, the training of this model required the greate number of steps and computational efforts. The model trained on 256 $\times$ 256 px images without augmentation (Model 20) has fewer false positive (FP) detection results than the model trained on 700 $\times$ 700 px images with augmentation (Model 50). Meanwhile, the model trained on 512 $\times$ 512 px images with augmentation (Model 30) also has fewer FP detection results than Model 50. It can be concluded that by increasing the training dataset, one can achieve a reduction in FP detection results for Model 50. Based on the data from Table 1, we concluded that Model 20 has the worst characteristics, and therefore, it was not further considered.

**Table 1:** Summarized parameters of the models trained in this study.

Model comparison			
Parameter	Model 20	Model 30	Model 50
Total steps	20,000	30,000	50,000
Warm steps	700	700	1000
Learning rate base	0.008	0.008	0.008
Image resize (px $\times$ px)	256 $\times$ 256	512 $\times$ 512	700 $\times$ 700
Augmentation	Only horizontal flip (after dataset formation)	All of the aforementioned	All of the aforementioned
True positive	16	16	15
False positive	5	3	6
False negative	29	29	30
Segmentation accuracy (dice coefficient)	0.61	0.68	0.72

**Figure 2:**
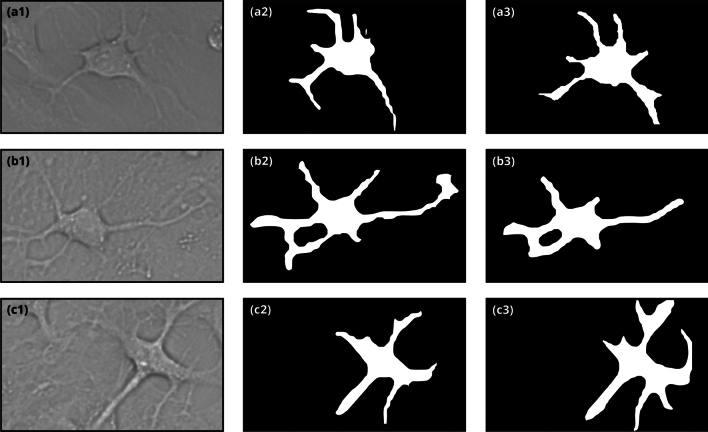
The region of interest (ROI) in the detection of the morphology of an astrocyte. The first column represents the image of an astrocyte, the second column represents the detection by the neural network Model 30, and the third column represents the detection by the neural network Model 50.

**Figure 3:**
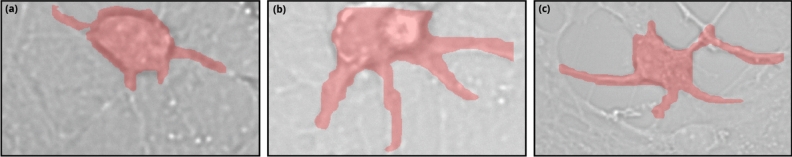
False positive (FP) results of astrocyte detection by Model 30. The segmented mask is highlighted in red.

The comparison between the segmentation obtained by models 30 and 50, using images that were not included in either the training or test sets, is depicted in Fig. 2. It can be observed that Model 50 detects a higher number of branches compared to Model 30, as evident in the second and third rows of Fig. 2. Additionally, in the second row of Fig. 2, the branches segmented by Model 30 are longer than those segmented by Model 50, but the masks have slight distortions along them. As previously discussed, this discrepancy is associated with the difference in the resolutions of the input images (512 $\times$ 512 pixels in Model 30 versus 700 $\times$ 700 pixels in Model 50) used during training. Note that although Model 50 achieves better segmentation, Model 30 exhibits superior performance in minimizing false positives results. It is important, since FP results contribute significant error to the overall statistics as they detect an area that was not labeled as an astrocyte. The high number of false negatives (FN) across all models (astrocyte was labeled but not detected) can be explained by potential errors in cell annotation. In Fig. 3, the model erroneously segmented the intercellular space instead of an astrocyte.

**Figure 4:**
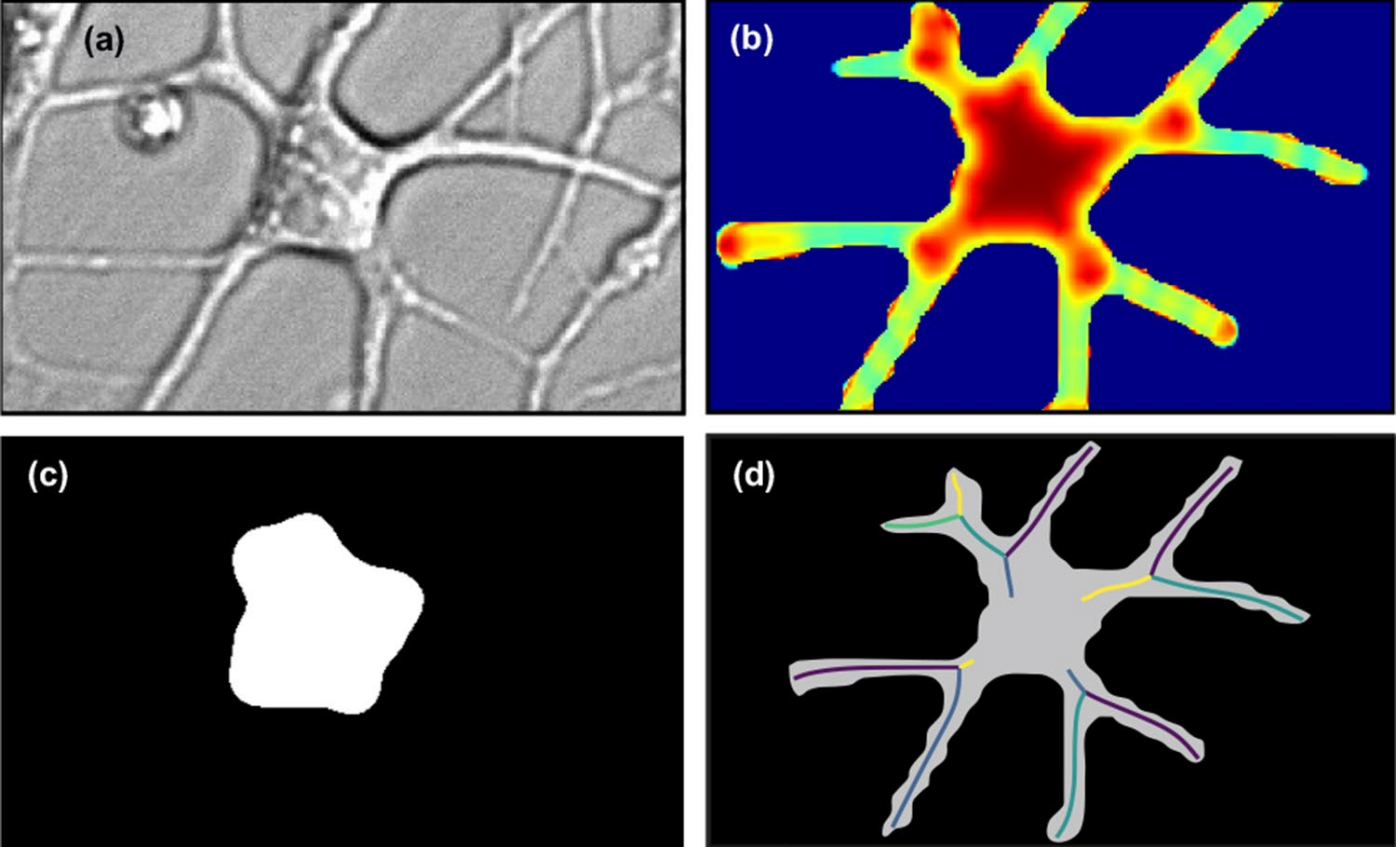
Segmentation of the soma in an astrocyte. The (**a**) is the original microscope image, the (**b**) is the image colored according to the directional ratio, the (**c**) is the segmented soma and the (**d**) is the segmented mask of an astrocyte and its skeletonized branches, different colors correspond to the recognized segments.

Then, we used Models 30 and 50 for post-processing all available experimental data to assess the morphological characteristics of isolated astrocytes during their time evolution. In particular, we focused on three characteristics: the total length of branches, the number of branchings, and the area of the astrocyte (including its branches). To detect astrocyte branches, we extract their soma based on the local isotropy of the image^88–90^. The directional ratio ranges from low values for vessel-like structures to 1 for drop-shaped structures. This approach has been shown to be very effective in separating soma from branches in fluorescent imaging of neurons^91^. In contrast to work^91^, we used masks obtained through a neural network rather than fluorescent images. Example of soma identification is shown in Fig. 4a–c.

After extracting the soma, we separated it from the whole astrocyte body, keeping only the branches. For this purpose, we subtracted the Boolean mask of the soma from the Boolean mask of the segmented astrocyte. The obtained branch masks underwent skeletonization using the scikit-image library function. The border pixels of the astrocyte branches were identified and removed under the condition that their removal did not disrupt the connectivity of the respective object. After skeletonization, the thickness of the astrocyte branch became one pixel (each pixel has two neighboring pixels except begining or end of the branch). Each branch consists of nodes and branch segments coming out of them. Search of segments was developed using a modernized deep-first search graph method: the number of neighboring pixels in skeletonized branch is sequentially calculated and in cases where the number of neighboring pixels exceeds 2 and the previous pixel is not a node, it indicates that this particular pixel serves as a node because more than one segment comes out from it, as shown in Fig. 4d. Note, if a border segment had a length less than 10 pixels (below the typical thickness of branches), we excluded it from consideration. The total length of the branch was calculated by summing the lengths of all segments between the nodes for each astrocyte. Based on this result, we identified nodes, branches and segments of the branches, allowing us to measure the dynamics of cell growth in the experiment.

**Figure 5:**
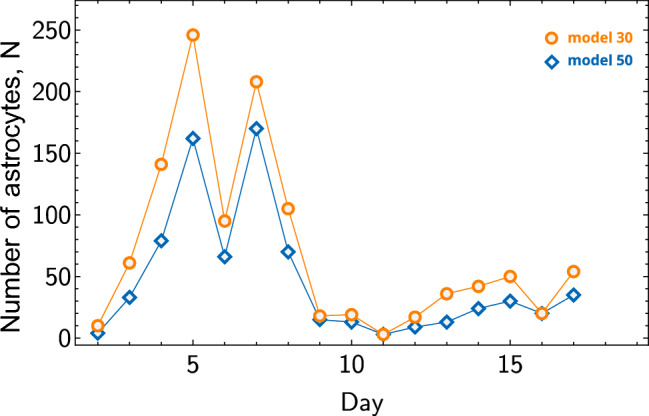
Number of detected astrocytes. Blue and orange symbols correspond to Models 50 and 30, respectively, with lines added for better visibility.

**Figure 6:**
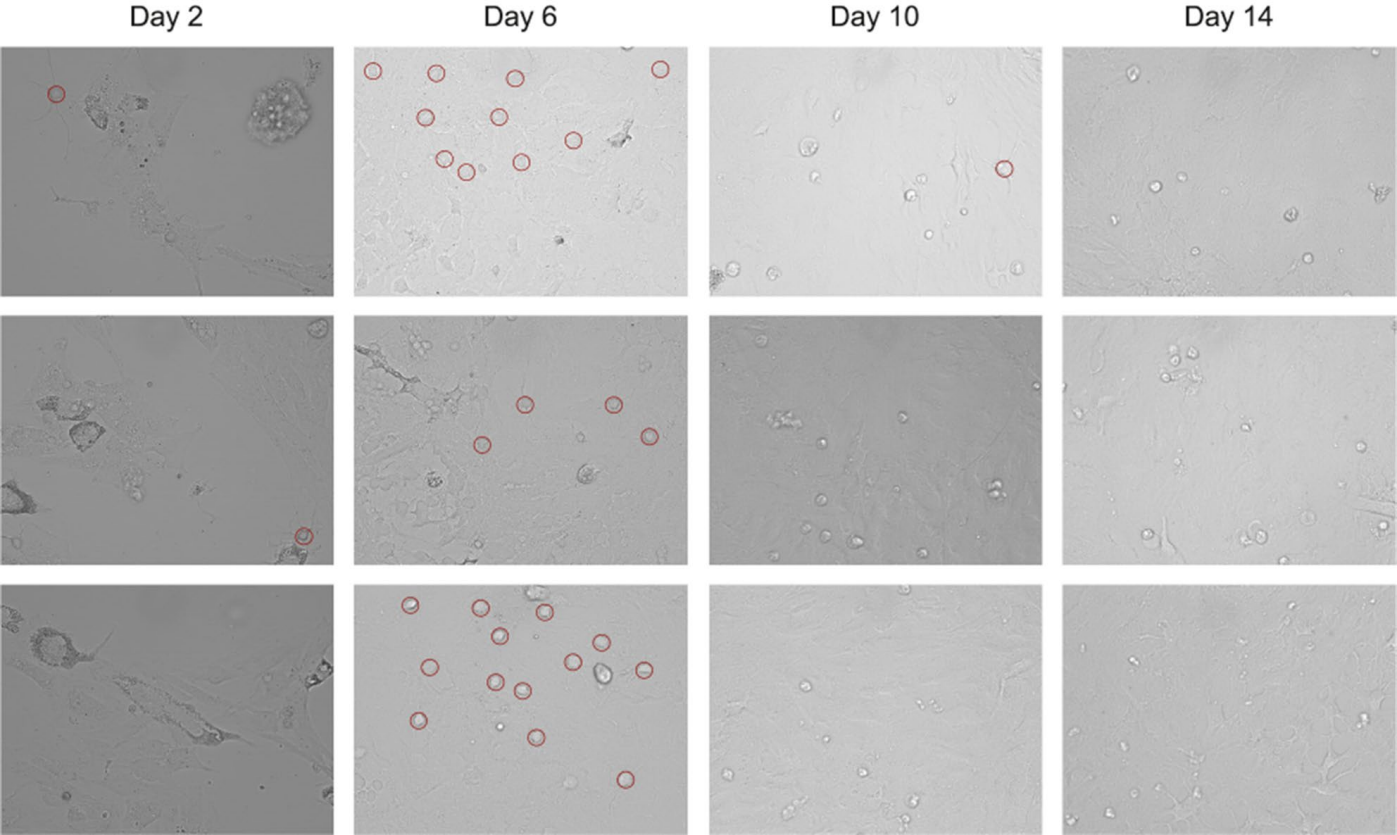
Images of astrocytes recorded on 2nd, 6th, 10th, 14th day respectively. The images represent a random set from microscopic data taken on the respective days. The red circles indicate the astrocytes that fell within the imaged area.

The dependency of the number of detected astrocytes by Models 30 and 50 in experimental images across different days of astrocyte cultivation are shown in Fig. 5. The maximum number of astrocytes is reached around days 5-6, but by day 10, the count of astrocytes significantly decreases. We attribute this to the fact that after the 6th day, astrocytes form a monolayer with a confluence of approximately 95%, that make them indistinguishable. Examples of a random set of experimental images obtained on different days are shown in Fig. 6.

**Figure 7:**
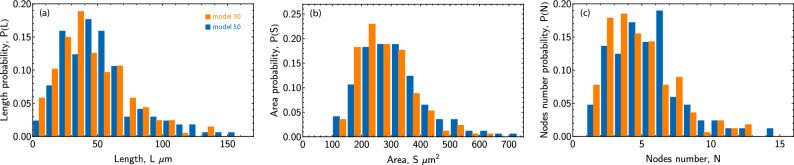
The histograms of astrocyte parameters, as measured on the 7th day of experiments. (**a**) The distribution by lengths, (**b**) the distribution by areas, and (**c**) the distribution by the number of nodes. The orange bars represent the results of Model 30, while the blue bars represent the results of Model 50. The touching blue and red bars refer to the same value.

**Figure 8:**
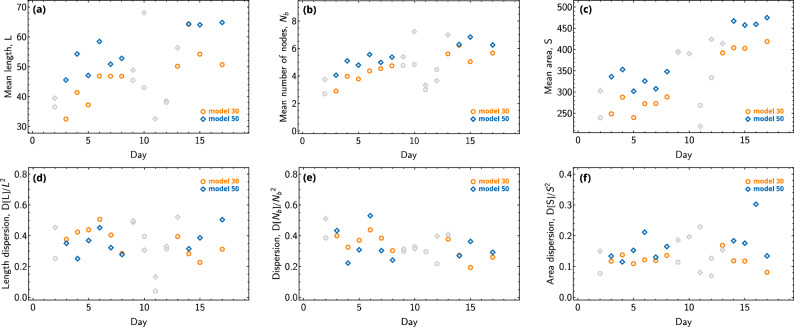
The temporal dependencies of astrocyte parameters are shown: (**a**) illustrates the average length of branches *L*, (**b**) shows the average number of nodes per astrocyte $N_b$, and (**c**) presents the average area *S* of the astrocyte. (**d**–**f**) The dependencies of dispersion of the considered characteristics, normalized to the mean value squared, $D[L]/L^2$, $D[N_b]/N_b^2$, and $D[S]/S^2$ respectively. The orange symbols represent the results of Model 30, while blue symbols represent the results of Model 50. Data points calculated from samples of fewer than 20 astrocytes are colored gray.

Histograms of the lengths of all astrocyte branches, astrocyte areas, and the number of nodes measured on the 7th day, during which both models allowed the detection of a sufficient number of astrocytes, are presented in Fig. 7. It can be noted that overall both models lead to similar distributions. Finally, Fig. 8. shows the temporal dependencies of the mean values of the parameters we measured: total length of astrocyte branches *L*, number of nodes $N_b$, area *S*, as well as the corresponding variances *D*[*L*], $D[N_b]$, and *D*[*S*]. Orange circles represent data obtained using Model 30, while blue diamonds represent data by Model 50, points measured from samples of fewer than 20 astrocytes are colored gray. Overall, it can be noted that Model 50 estimates longer branches, recognizes a greater number of nodes, and consequently identifies a larger area of the astrocyte (which is considered along with the area of its branches). As noted earlier, this is because Model 50 segments astrocytes with high accuracy. At the same time, both models provide similar values of the investigated parameters dispersion. Important to note that the lengths and number of branchings we measure are notably lower compared to those measured when using cell staining^92^. This is due to limitations imposed by phase contrast microscopy, which does not allow resolving branches with a diameter of less than $\lesssim 0.8$ micrometers, consequently restricting the observation of smaller branching networks. The question of the possibility of reconciling data from phase contrast microscopy with fluorescent microscopy of stained cells, as well as the development of an extrapolative model to estimate the total length of branches based on phase contrast microscopy data, deserves further study. The values presented in Fig. 8 are summarized in Table 2 for Model 30 and in Table 3 for Model 50.

**Table 2:** The tabulated values of the parameters presented in Fig. 8 and calculated using Model 30.

Distribution parameters, Model 30						
Number of the day	Mean length, M[L], $\upmu$m	Length dispersion, D[L]/$L^2$	Mean branch nodes, M[$N_b$], $\upmu$m	Branch nodes dispersion, D[$N_b$]/$N_{b}^2$	Mean area, M[S], $\upmu m^2$	Area dispersion, D[S]/$S^2$
2	36.54	0.2516	2.70	0.3855	241.76	0.0658
3	32.50	0.3773	2.90	0.3999	247.70	0.1130
4	41.40	0.4241	3.98	0.3257	282.01	0.1530
5	37.25	0.4386	3.78	0.3704	243.82	0.1100
6	46.87	0.5066	4.37	0.4380	271.25	0.0998
7	46.82	0.4052	4.54	0.3846	275.25	0.1200
8	46.83	0.2850	4.74	0.3032	279.72	0.1210
9	45.47	0.4884	4.78	0.2996	390.09	0.1100
10	43.01	0.3956	4.84	0.3289	367.22	0.1900
11	25.74	0.0403	3.00	0.2963	323.78	0.0942
12	38.50	0.3148	4.47	0.2185	333.82	0.1270
13	50.17	0.3953	5.61	0.3781	376.97	0.1800
14	64.27	0.2832	6.24	0.2727	392.93	0.1230
15	54.23	0.2269	5.04	0.1936	393.28	0.1160
16	53.05	0.3015	5.65	0.2515	433.39	0.1320
17	50.70	0.3123	5.67	0.2607	442.24	0.0758

**Table 3:** The tabulated values of the parameters presented in Fig. 8 and calculated using Model 50.

Distribution parameters, Model 50						
Number of the day	Mean length, M[L], $\upmu$m	Length dispersion, D[L]/$L^2$	Mean branch nodes, M[$N_b$], $\upmu$m	Branch nodes dispersion, D[$N_b$]/$N_{b}^2$	Mean area, M[S], $\upmu m^2$	Area dispersion, D[S]/$S^2$
2	39.47	0.4540	3.75	0.5111	327.09	0.1100
3	45.58	0.3508	4.06	0.4335	334.39	0.1190
4	54.31	0.2511	5.10	0.2224	345.26	0.1090
5	47.09	0.3691	4.79	0.3085	306.27	0.1420
6	58.47	0.4524	5.56	0.5303	329.11	0.1440
7	50.88	0.3230	4.99	0.3035	309.91	0.1270
8	52.81	0.2777	5.37	0.2418	337.92	0.1310
9	48.85	0.4969	5.40	0.3146	384.74	0.1660
10	68.12	0.3063	7.23	0.3153	450.27	0.1260
11	32.60	0.1334	3.33	0.0200	219.12	0.0810
12	38.06	0.3319	3.67	0.3967	424.16	0.0702
13	56.35	0.5203	7.00	0.4082	414.26	0.1540
14	64.32	0.3154	6.29	0.2705	466.93	0.1840
15	64.07	0.3867	6.83	0.3628	459.02	0.1650
16	54.66	0.3816	5.75	0.3807	468.14	0.2660
17	64.82	0.5046	6.26	0.2924	530.76	0.1360

For in vitro studies, the method should be sensitive enough to various changes in cell morphology induced by the local microenvironment (such as stressful conditions or paracrine and autocrine signaling). To demonstrate the ability of our approach to detect such effects, we run a special series of experiments with Nicotinamide as a well-known modulator of intracellular NAD+ metabolism. Physiology and morphology of astrocytes could be easily affected by altered NAD+ levels (e.g. in aging, ischemia, or neurodegeneration) because glycolytic activity and NAD+-dependent enzymes control their activation, migration and interactions with neurons. Nicotinamide (0.1 mmol) was added to astrocytes on the second day of their culture. Morphological parameters of astrocytes at different time points of nicotinamide exposure are presented in Table 4 for Model 30 and in Table 5 for Model 50. Note that our method allows us to identify noticeable deviations in the mean values and variances of the tested morphological characteristics in the astroglial culture exposed to Nicotinamide.

**Table 4:** The tabulated values of the parameters after nicotinamide addition calculated using Model 30.

Distribution parameters, Model 30						
Number of the day	Mean length, M[L], $\upmu$m	Length dispersion, D[L]/$L^2$	Mean branch nodes, M[$N_b$], $\upmu$m	Branch nodes dispersion, D[$N_b$]/$N_{b}^2$	Mean area, M[S], $\upmu m^2$	Area dispersion, D[S]/$S^2$
2	71.71	0.1998	1.00	0.0000	372.10	0.1168
3	62.91	0.0663	1.00	0.0000	301.00	0.0786
4	48.20	0.0000	1.00	0.0000	318.05	0.0000
5	100.37	0.0660	0.00	0.0000	497.73	0.0389
6	53.98	0.0429	0.00	0.0000	256.15	0.1041
7	92.40	0.1394	1.50	0.0494	436.06	0.0766
8	78.16	0.04943	1.00	0.0000	441.23	0.0552
9	57.64	0.2668	0.00	0.0000	327.14	0.0192
10	60.76	0.3004	2.00	0.0000	495.03	0.0423
11	29.11	0.1803	0.00	0.0000	315.42	0.0555

**Table 5:** The tabulated values of the parameters after nicotinamide addition calculated using Model 50.

Distribution parameters, Model 50						
Number of the day	Mean length, M[L], $\upmu$m	Length dispersion, D[L]/$L^2$	Mean branch nodes, M[$N_b$], $\upmu$m	Branch nodes dispersion, D[$N_b$]/$N_{b}^2$	Mean area, M[S], $\upmu m^2$	Area dispersion, D[S]/$S^2$
2	70.08	0.1506	1.00	0.0000	373.26	0.0552
3	64.67	0.0873	2.00	0.0000	275.57	0.1146
4	–	–	–	–	–	–
5	99.81	0.0054	1.00	0.0000	528.81	0.1024
6	–	–	–	–	–	–
7	110.35	0.0997	2.00	0.0000	552.09	0.1205
8	107.41	0.0559	1.00	0.0000	431.73	0.1020
9	45.45	0.0791	0.00	0.0000	281.46	0.0384
10	29.95	0.0000	0.00	0.0000	521.52	0.0000
11	33.26	0.1325	1.00	0.0000	266.84	0.1161

It is worth noting that the problem of analyzing the evolution of morphological parameters has also been addressed in other studies, for example in^93^, where the NeuriTES platform was developed to track the dynamic evolution of neurons. This method requires manually labeling the neuron whose evolution is to be tracked and using a measure of transfer entropy to study the relationships between parameters relevant to the evolving system. In contrast, in our work, we proposed to collect statistics of morphological features by days. That is, by imaging a large region, automatically recognizing astrocytes, and extracting morphological parameters of interest.

## Conclusion

We performed the experiments with primary cultures of rat brain cortical astrocytes visualized with phase contrast microscopy at different time points of their growth in vitro. We labeled the data and trained three models based on deep convolutional neural networks that enable the recognition of astrocytes in low-contrast images (obtained without using cell staining) and the analysis of their morphology. Using the obtained models, we measured how morphological characteristics such as total branch length, branching count, and overall size of an astrocyte are changed along their growth in the culture. The results of our study show that the application of a neural network to process experimental data with the study of astrocyte growth dynamics can significantly improve the speed of analysis. The novelty of the proposed approach lies in its ability to recognize morphological data from unstained cell microscopic images. Our approach allows us to automate the data processing, making it more efficient. We have demonstrated that training of the neural network with the data obtained in samples that have been thoroughly analyzed by qualified neuroscientists allows achieving good results in predicting the outcomes of astroglia branching. Our results significantly extend the potential use of neural networks in neurobiology and represent an important contribution to the development of experimental data processing methods in this field.

In the conclusion, it is possible to formulate a series of problems and directions for further studies, in which the obtained results and methods can serve as a basis. Firstly, by augmenting the training dataset with data containing a priori known other cell types, the proposed approach can be generalized for the recognition of various cell types in phase-contrast microscopy data. Furthermore, having sufficient datasets for cell morphology analysis based on phase contrast and fluorescence methods, it may be possible to establish accurate correspondences between them, potentially speeding up and reducing the cost of a range of cell morphology studies by eliminating the need for staining. Furthermore, when properly generalized and integrated with a system for automatic periodic scanning of plates with cell culture, our method can enable the development of a system capable of tracking and analyzing the morphology of each individual cell during its cultivation. Finally, the analysis of three-dimensional cellular structures is of great interest, which requires the development of separate methods and algorithms. One possible approach to developing such methods is the synthesis of computer-generated 3D structures based on a series of two-dimensional images, which is actively used in solving similar tasks in the field of soft matter physics. It is worth noting that the proposed method, as well as possible extensions to 3D structures, tracking individual cells, and identifying their types, will be useful for the development of digital twins of individual cells and organoids. For example, these digital twins could be based on cellular Potts models and implemented in packages such as CompuCell3D or other agent-based approaches. In particular, such models require significant statistics of the morphological parameters of cells under different conditions for parameterizing the models or their final verification. In turn, digital twins will allow us to better understand the mechanisms of various processes occurring in cell systems, as well as reduce the number of experiments. All of the above indicates the potential benefit of the proposed method for the scientific communities in biology, biophysics, medicine, neuroscience, and bioimaging.

## Materials and methods

### Isolation and cultivation of astrocytes from the rat brain cortex

A dissociated culture of astrocytes was obtained from newborn Wistar rats using the method of enzymatic-mechanical dissociation according to a previously described method^94,95^. The isolated structures were rinsed with calcium and magnesium-free phosphate-buffered saline (PBS, pH 7.4, Gibco Life Technologies, USA), minced with a scalpel, and incubated for 15 min at 37 ^∘^C in a 0.05$\%$ trypsin and 0.02$\%$ EDTA solution (Gibco Life Technologies, USA). After two washes with phosphate buffer, the structures were mechanically dissociated in culture medium using stepwise pipetting. The culture medium consisted of 90$\%$ minimum essential medium (MEM Gibco, UK), 10$\%$ fetal bovine serum (FBS), 2 mM glutamax (Gibco, UK), 10 mM HEPES buffer (Sigma, USA), pH 7.2–7.4. The cell suspension was centrifuged for 3 minutes at 1000 rpm, the pellet was resuspended in the culture medium, and cells were seeded into two T-25 culture flasks. Cultures were maintained in a CO_2_ incubator (RWD Life Science, China) at 37 ^∘^C, 5$\%$ CO_2_, and 98$\%$ relative humidity. On the second day of cultivation, a complete change of the culture medium was performed to a medium containing 10% FBS. Upon monolayer formation, astrocytic cultures were transferred to 96-well plastic plates (Servicebio, China) with 100 $\upmu$l of a suspension containing $2\times 10^6$ cells per ml. This two actions also serves as a step in purifying the astrocyte culture. Cell counting and viability assessment were performed using the Countess automated cell counter (Invitrogen), which recorded total cell count, live and dead cells, survival percentage, and cell size distribution. All experimental protocols were approved by the Ethical Committee of the “Research Center of Neurology”.

### Immunofluorescence assay

The isolated primary culture cells were transferred onto cover slides and incubated in a 10$\%$ FBS culture medium upon monolayer formation. The media was discarded, and cells were washed in PBS. The cells were further fixed with fresh 5$\%$ paraformaldehyde (PFA) for 15 min, samples were treated with 0.1$\%$ TritonX-100 (Calbiochem Biochemicals, USA) and blocked with 5$\%$ bovine serum albumin (BSA, Sigma, Germany). The primary antibodies were diluted in the IHCDiluent (Leica), placed on cover slides, incubated at 4 ^∘^C for 6 h. The selected antibodies were rabbit anti-GFAP antibody (1:250, Abcam, ab68428), rabbit anti-NeuN antibody (1:200, ABclonal, A19086), rabbit anti- IBA1 antibody (1:200, Affinity Biosciences, DF6442), rabbit anti-PDGFRB antibody (1:300, Elabscience, E-AB-32531). After five washes with PBS, the cells were incubated with Goat Anti-Rabit IgG(H+L) Alexa Fluor 594-cojugated (1:250, Elabscience, E-AB1060) or Alexa Fluor 594-conjugated Donkey Anti-Rabit IgG(H+L) (1:200, Fine Test, FNSA-0063), FITC Goat Anti-Rabbit IgG (H+L) (1:200, ABclonal, AS011) or FITC-linked Guinea pig Anti-Rabbit IgG Polyclonal antibody (1:1000, Cloud-Clone, SAA544Rb58) secondary antibodies for 2 h at room temperature. Then cells were washed with PBS twice and stained with Fluoroshield and DAPI (Sigma). The slides proceeded without primary antibodies were used as a negative control. Images were captured using EVOS M7000 system (ThermoFisher Scientific).

### Identification of culture cells

**Figure 9:**
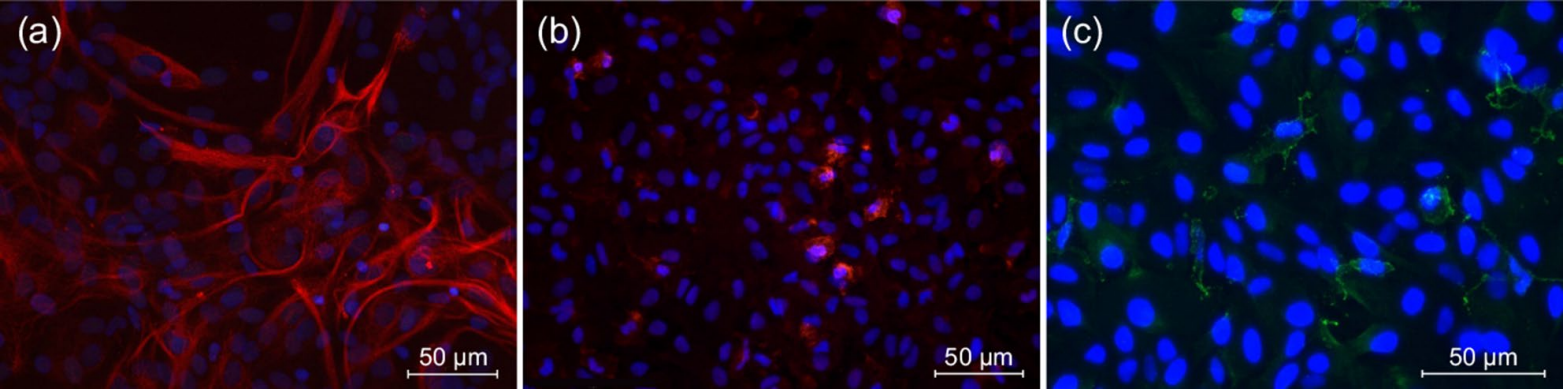
Identification of culture cells: (**a**) astrocytes in the primary culture: the cell bodies and processes are stained with GFAP antibody (red), the nuclei are stained with DAPI (blue), (**b**) microglia in the primary culture: cell bodies and processes are stained with Iba1 antibody (red), the nuclei are stained with DAPI (blue), (**c**) pericytes in the primary culture: the cell bodies and processes are stained with PDGFRB antibody (green), the nuclei are stained with DAPI (blue).

Immunophenotyping of neuronal, astroglial and microglial cells was performed to confirm the purity of the primary culture. As shown in Fig. 9a, the majority of isolated cells was positively stained with the astrocyte marker GFAP. Negative results have been obtained in immunostaining with NeuN antibodies that recognize mature neurons, thereby confirming the absence of neuronal cells in the primary culture. The cells in the primary culture were positively stained with a microglial marker Iba1 (Fig. 9b), but not more than 10$\%$ of Iba1-immunopositive cells have been found. Also, cells in the primary culture were positively stained with a pericyte marker PDGFRB, the number of PDGFRB-positive cells was no more than 8$\%$ (Fig. 9c). Thus, we confirm that astrocytes are the predominant type of cells within the primary culture which was further used for the application of machine learning algorithms to recognize astrocytes in phase-contrast images.

### Phase-contrast microscopy

**Figure 10:**
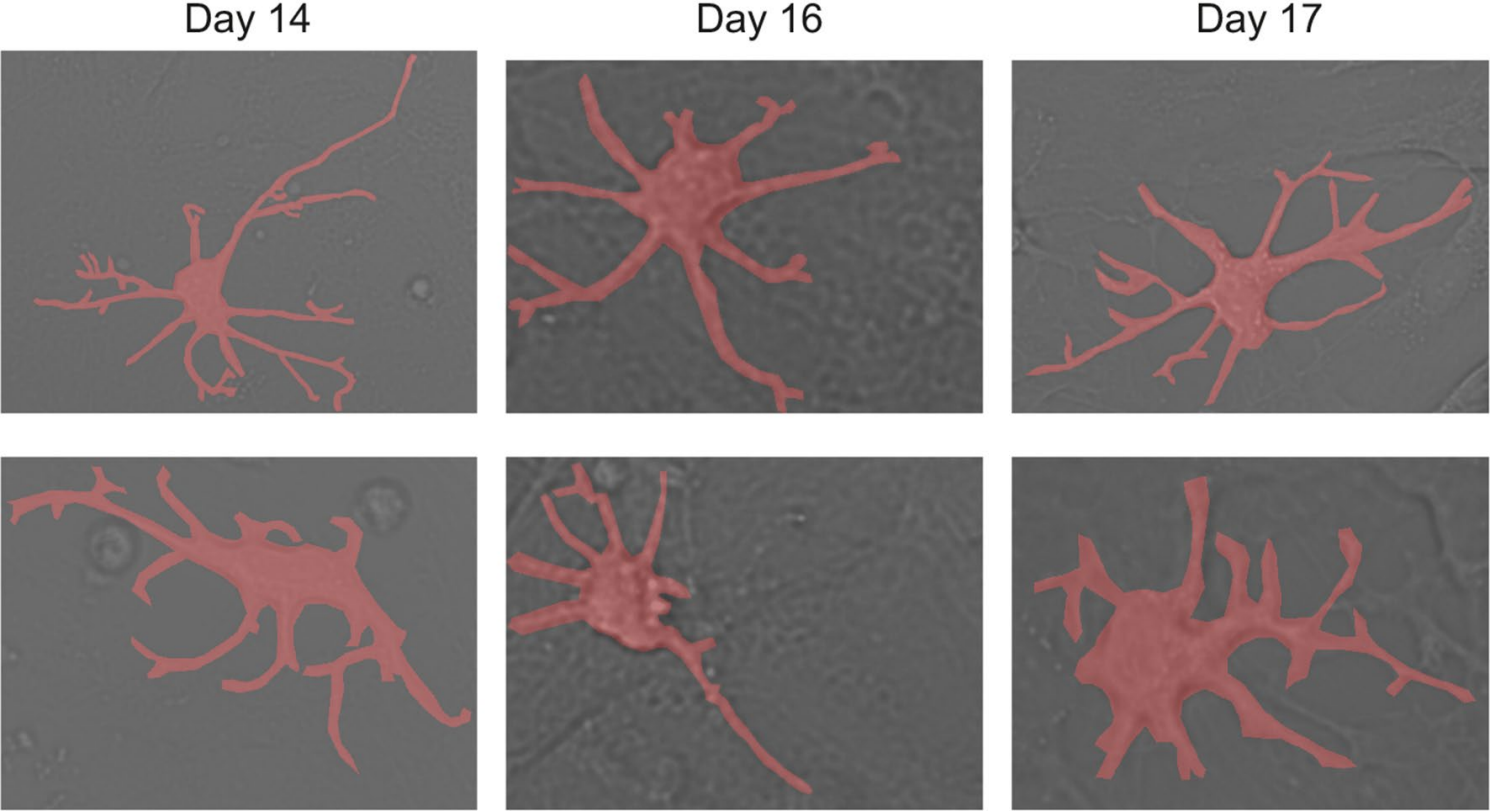
Images acquired during the same experiment (on the same day and on an identical setup) to check for possible bias between experiments under nominally identical conditions.

To study morphology of astrocytes in their native state, we applied the phase contrast microscopy which does not require any staining and fixation. Live observations were conducted using phase-contrast microscopy with the “EVOS M7000” imaging system (Thermo Fisher Scientific, USA). Experimental work was carried out to visualize rat astrocyte cells during their growth. An EVOS M7000 phase contrast microscope with an AMC1000 stage incubator was used for observation. A 96-well plate with cells was installed inside a built-in incubator with motorized stages. We conducted a series of experiments over a duration of 18 days. Microphotography of cells was carried out daily and included photographic recording of a 4x4 frame field in each well of the plate at a magnification of 40x starting from the second day of cultivation. Note that the study area was selected randomly, without preliminary analysis for the presence of astrocytes. Since the microscope has motorized stage movers, it was possible to track the position of the frame according to coordinates in the XY plane throughout the entire well area. This approach allowed the same area of each well to be recorded daily. Thus, as a result of the experiments, photographs of astrocytes were obtained at different moments of their growth. The experimental work involved capturing photographic records of astrocytes through microscopy of 6 wells within the plate. In total, over 1100 astrocytes were recorded at various growth stages. The results of the experiment are low-contrast images of astrocytes, which are subsequently the object of analysis for neural networks. In Fig. 10. we show images acquired during the same experiment (on the same day and on an identical setup) to check for possible bias between experiments under nominally identical conditions.

## Data Availability

The data that support the findings of this study are available from the corresponding author upon reasonable request.
